# The Numerical Semigroup of Phrases' Lengths in a Simple Alphabet

**DOI:** 10.1155/2013/459024

**Published:** 2013-11-19

**Authors:** Aureliano M. Robles-Pérez, José Carlos Rosales

**Affiliations:** ^1^Departamento de Matemática Aplicada, Universidad de Granada, 18071 Granada, Spain; ^2^Departamento de Álgebra, Universidad de Granada, 18071 Granada, Spain

## Abstract

Let *𝒜* be an alphabet with two elements. Considering a particular class of words (the phrases) over such an alphabet, we connect with the theory of numerical semigroups. We study the properties of the family of numerical semigroups which arise from this starting point.

## 1. Introduction

Let *𝒜* be a nonempty finite set called the *alphabet*. Elements of *𝒜* are called *letters* or *symbols*. A *word* is a sequence of letters, which can be finite or infinite. We denote by 𝒜* (resp., *𝒜* ^*ω*^) the set of all finite (resp., infinite) words over *𝒜*. The sequence of zero letters is called the *empty word* and is denoted by *ɛ*. Any subset *ℒ*⊆𝒜* is called a *language* over *𝒜*. The *length* of a word *u* is denoted by |*u*|. If *u*, *v* are words, we define their *product* or *concatenation* as the word *uv*. We say that a word *u* is a *factor* of a word *v* if there exist two words *x*, *y* such that *v* = *xuy*. If *u* is a factor of *v* with *x* = *ɛ* (resp., *y* = *ɛ*), then *u* is a *prefix* (resp., *suffix*) of *v*.

We have taken these definitions from [1]. In this book (and the references given therein), the authors study problems related to Combinatorics on Words. However, we are going to consider a different point of view. We are interested in a very particular type of words (the phrases) and, more specifically, their length.

**Definition**

Let us take *𝒜* = {*a*, ⌣}. We say that *f* ∈ 𝒜* is a *phrase* if it fulfills the following conditions:

1. ⌣ is not a prefix or suffix of *f*,
2. ⌣⌣ is not a factor of *f*.

We denote *𝒜* ^*ℱ*^ = {*f* ∈ 𝒜* | *f* is a phrase}.

If we consider that ⌣ represents a gap between two words, then we have a suitable justification for the above definition.

Let *𝒞* be a language over *𝒜* such that *𝒞*⊆*𝒜* ^*ℱ*^. We will denote by *ℓ*(*𝒞*) = {|*c*| | *c* ∈ *𝒞*}. In this work we are going to deal with the structure of the set *ℓ*(*𝒞*) for particular choices of *𝒞*. In fact, let {*w*_1_,…, *w*_*n*_}⊆𝒜* be a finite set of words such that ⌣ is not a factor of *w*_*i*_,  1 ≤ *i* ≤ *n*. Then *𝒞*(*w*_1_,…, *w*_*n*_)⊆*𝒜* ^*ℱ*^ is the language in which each phrase *f* is obtained as product of factors belonging to {*w*_1_,…, *w*_*n*_}∪{⌣}. Moreover, in order to achieve the results of this paper, we assume that *ɛ* ∈ *𝒞*(*w*_1_,…, *w*_*n*_).

**Example**

If we take {*aaaa*, *aaaa* *a*}, then *f*_1_ = *aaaa* *aaaa*, *f*_2_ = *aaaa* *aaaa* *a*, and *f*_3_ = *aaaa* *a*_⌣_ *aaaa* belong to *𝒞*(*aaaa*, *aaaa* *a*). However, *f*_4_ = *aaaa*_⌣_ *aaaa* *aa*, *f*_5_ = *aaa*, and *f*_6_ = *aa*_⌣_ *a* do not belong to *𝒞*(*aaaa*, *aaaa* *a*).

Let *ℕ* be the set of nonnegative integers. A *numerical semigroup* is a subset *S* of (*ℕ*, +) that is closed under addition, contains the zero element, and such that *ℕ*∖*S* is finite.

In Section 2 we will show that *ℓ*(*𝒞*(*w*_1_,…, *w*_*n*_)) is a numerical semigroup. We will also see that there exist numerical semigroups that cannot be obtained by this procedure. This fact allows us to give the following definition.

**Definition**

Let *𝒜* be the alphabet given by the set {*a*, ⌣}. A numerical semigroup *S* is the *set of lengths of a language of phrases* (PL-semigroup for abbreviation) if there exists *𝒞* = *𝒞*(*w*_1_,…, *w*_*n*_)⊆*𝒜* ^*ℱ*^ such that *S* = *ℓ*(*𝒞*).

The next aim of Section 2 will be to characterize PL-semigroups. Concretely, we will show that a numerical semigroup *S* is a PL-semigroup if and only if *x* + *y* + 1 ∈ *S* for all *x*, *y* ∈ *S*∖{0}.

Let *S* be a numerical semigroup. Since *ℕ*∖*S* is a finite set, we can consider two notable invariants of *S* (see [2]). On the one hand, the *Frobenius number* of *S* is the maximum of *ℕ*∖*S* and is denoted by F(*S*). On the other hand, the *genus* of *S* is the cardinality of *ℕ*∖*S* and is denoted by g(*S*).

A *Frobenius variety* is a nonempty family *𝒱* of numerical semigroups that fulfills the following conditions:

1. if *S*, *T* ∈ *𝒱*, then *S*∩*T* ∈ *𝒱*,
2. if *S* ∈ *𝒱* and *S* ≠ *ℕ*, then *S* ∪ {F(*S*)} ∈ *𝒱*.

Let us denote *𝒮*_PL_ = {*S* | *S* is a PL-semigroup}. In Section 3, we will show that *𝒮*_PL_ is a Frobenius variety. This fact, together with the results of [3], will allow us to show orderly the elements of *𝒮*_PL_ in a tree with root *ℕ*. Moreover, we will also characterize the sons of a vertex, in order to build recursively such a tree.

The *multiplicity* of a numerical semigroup *S*, denoted by m(*S*), is the minimum of *S*∖{0}. We will study the set *𝒮*_PL_(*m*) = {*S* ∈ *𝒮*_PL_ | m(*S*) = *m*} in Section 4. In particular, we will show that this set is finite and has maximum and minimum with respect to the inclusion order. Furthermore, we will determine the sets {F(*S*) | *S* ∈ *𝒮*_PL_(*m*)} and {g(*S*) | *S* ∈ *𝒮*_PL_(*m*)}. We will also see that the elements of *𝒮*_PL_(*m*) can be ordered in a tree with root the numerical semigroup *S* = {0, *m*, →} (where the symbol → means that every integer greater than *m* belongs to *S*).

In Section 5, we will see that a PL-semigroup is determined perfectly by a nonempty finite set of positive integers. In addition, we will show explicitly the smallest PL-semigroup that contains a given nonempty finite set of positive integers.

We finish this introduction pointing out that this work admits different generalizations. Some of them are in working process and other ones have already been developed (see [4]).

## 2. PL-Semigroups

If *X* is a nonempty subset of *ℕ*, we denote by 〈*X*〉 the submonoid of (*ℕ*, +) generated by *X*; that is,

(1) 〈X〉={λ1x1+⋯+λnxn ∣ n∈ℕ∖{0}, x1,…,xn∈X, λ1,…,λn∈ℕ}.

It is a well-known fact (see for instance [5, Lemma 2.1]) that 〈*X*〉 is a numerical semigroup if and only if gcd{*X*} = 1 (as usual, gcd means *greatest common divisor*). On the other hand, every numerical semigroup *S* is finitely generated, and therefore there exists a finite subset *X* of *S* such that *S* = 〈*X*〉. In addition, if no proper subset of *X* generates *S*, then we say that *X* is a *minimal system of generators* of *S*. In [5, Theorem 2.7] it is proved that every numerical semigroup *S* has a unique (finite) minimal system of generators. The elements of such a system are called *minimal generators* of *S*.

Let *𝒜* be an alphabet and let {*w*_1_,…, *w*_*n*_}⊆𝒜* be a finite set of words. If *𝒞* = *𝒞*(*w*_1_,…, *w*_*n*_)⊆𝒜* is the language in which each word is obtained as product of factors belonging to {*w*_1_,…, *w*_*n*_}, then it is easy to see that *ℓ*(*𝒞*) is a submonoid of (*ℕ*, +). In addition, if gcd{|*w*_1_|,…, |*w*_*n*_|} = 1, then *ℓ*(*𝒞*) is a numerical semigroup. Moreover, it is a simple exercise to show that we can get any numerical semigroup in this way.

As we indicated in the introduction we are going to study the particular case in which we consider lengths of phrases. Consequently, we will focus our attention in a particular family of numerical semigroups.

**Proposition**

Let *𝒜* = {*a*, ⌣} be an alphabet. If *𝒞* = *𝒞*(*w*_1_,…, *w*_*n*_)⊆*𝒜* ^*ℱ*^, then *ℓ*(*𝒞*) is a numerical semigroup.

**Proof**

We proceed in three steps.

- First of all, being that *ɛ* ∈ *𝒞* and |*ɛ*| = 0, we have that 0 ∈ *ℓ*(*𝒞*).
- Now, let us see that, if *l*_1_, *l*_2_ ∈ *ℓ*(*𝒞*), then *l*_1_ + *l*_2_ ∈ *ℓ*(*𝒞*). In effect, let *f*_1_, *f*_2_ ∈ *𝒞* such that |*f*_1_| = *l*_1_ and |*f*_2_| = *l*_2_. Then the concatenation *f*_1_ *f*_2_ (of *f*_1_ and *f*_2_) is an element of *𝒞* with |*f*_1_ *f*_2_| = *l*_1_ + *l*_2_.
- Finally, let *f* ∈ *𝒞* with |*f*| ≠ 0 (i.e., *f* ≠ *ɛ*). Since *f*_⌣_ *f* ∈ *𝒞*, we have that {|*f*|, 2|*f*| + 1}⊆*ℓ*(*𝒞*). By the previous step, we know that *𝒞* is closed under addition and, consequently, 〈|*f*|, 2|*f*| + 1〉⊆*ℓ*(*𝒞*). As gcd{|*f*|, 2|*f*| + 1} = 1, we have 〈|*f*|, 2|*f*| + 1〉 is a numerical semigroup. Therefore, *ℕ*∖*ℓ*(*𝒞*) is finite.

We conclude that *ℓ*(*𝒞*) is a numerical semigroup.

From now on, unless another thing is stated, we take *𝒜* = {*a*, ⌣}. As in the introduction, we say that a numerical semigroup *S* is a PL-semigroup if there exists *𝒞* = *𝒞*(*w*_1_,…, *w*_*n*_)⊆*𝒜* ^*ℱ*^ such that *S* = *ℓ*(*𝒞*). From Proposition 4, we deduce that if *S* is a PL-semigroup and *x* ∈ *S*∖{0}, then 2*x* + 1 ∈ *S*. Consequently, there exist numerical semigroups which are not of this type. For example, *S* = 〈5,7, 9〉 is not a PL-semigroup because 2 · 5 + 1 = 11 ∉ *S*.

In the next result we give a characterization of PL-semigroups.

**Theorem 5**

Let *S* be a numerical semigroup. The following conditions are equivalent.

1. *S* is a *PL*-semigroup.
2. If *x*, *y* ∈ *S*∖{0}, then *x* + *y* + 1 ∈ *S*.

**Proof**

(1⇒2) By hypothesis, *S* = *ℓ*(*𝒞*(*w*_1_,…, *w*_*n*_)) for some nonempty finite set {*w*_1_,…, *w*_*n*_}⊆𝒜*. If *x*, *y* ∈ *S*∖{0}, then there exist *f*, *g* ∈ *𝒞*(*w*_1_,…, *w*_*n*_)∖{*ɛ*} such that |*f*| = *x* and |*g*| = *y*. It is clear that *f*_⌣_ *g* ∈ *𝒞*(*w*_1_,…, *w*_*n*_) and |*f*_⌣_ *g*| = *x* + *y* + 1. Therefore, *x* + *y* + 1 ∈ *S*.

(2⇒1) Let {*n*_1_,…, *n*_*p*_} be the minimal system of generators of *S*. Let us take the set {*w*_1_,…, *w*_*p*_ | |*w*_*i*_| = *n*_*i*_,  1 ≤ *i* ≤ *p*}. Our aim is to show that if *𝒞* = *𝒞*(*w*_1_,…, *w*_*p*_), then *S* = *ℓ*(*𝒞*).

Since {*n*_1_,…, *n*_*p*_}⊆*ℓ*(*𝒞*), by applying Proposition 4, we have *S* = 〈*n*_1_,…, *n*_*p*_〉⊆*ℓ*(*𝒞*). Now, let *l* ∈ *ℓ*(*𝒞*). In order to prove that *l* ∈ *S*, we are going to use induction over *l*. If *l* = 0, then the result is trivially true. Let us assume that *l* > 0, and let *f* ∈ *𝒞* such that |*f*| = *l*. If ⌣ is not a factor of *f*, then the result follows immediately. In other case, there exist *f*_1_, *f*_2_ ∈ *𝒞*∖{*ɛ*} such that *f* = *f*_1⌣_ *f*_2_. By hypothesis of induction, |*f*_1_|, |*f*_2_| ∈ *S*. Thereby, *l* = |*f*| = |*f*_1_| + |*f*_2_| + 1 ∈ *S*.

**Remark 6**

The previous theorem leads to the concept of *numerical semigroup that admit a linear nonhomogeneous pattern*. For a general study of this family of numerical semigroups see, for instance, [6, 7].

Let *S* be a numerical semigroup with minimal system of generators given by {*n*_1_,…, *n*_*p*_}. Following [8], if *s* ∈ *S*, then we define the *order* of *s* (in *S*) by

(2) ord(s;S)=max⁡{α1+⋯+αp ∣ α1n1+⋯+αpnp=s,with α1,…,αp∈ℕ}.

If no ambiguity is possible, then we write ord(*s*).

**Remark 7**

From [5, Lemma 2.3 and Theorem 2.7], we have that if *X* is the minimal system of generators of *S*, then every system of generators of *S* contains *X*. Consequently, the definition of ord(*s*; *S*) does not depend on the considered system of generators; that is, it only depends on *s* and *S*.

**Lemma 8**

Let *S* be a numerical semigroup with minimal system of generators given by {*n*_1_,…, *n*_*p*_} and let *s* ∈ *S*.

1. If *i* ∈ {1,…, *p*} and *s* − *n*_*i*_ ∈ *S*, then ord(*s* − *n*_*i*_) ≤ ord(*s*) − 1.
2. If *s* = *α*_1_ *n*_1_ + ⋯+*α*_*p*_ *n*_*p*_, with ord(*s*) = *α*_1_ + ⋯+*α*_*p*_ and *α*_*i*_ ≠ 0, then ord(*s* − *n*_*i*_) = ord(*s*) − 1.

**Proof**

(1) Assume that *s* − *n*_*i*_ = *β*_1_ *n*_1_ + ⋯+*β*_*p*_ *n*_*p*_, with *β*_1_ + ⋯+*β*_*p*_ = ord(*s* − *n*_*i*_). Then *s* = *β*_1_ *n*_1_ + ⋯+(*β*_*i*_ + 1)*n*_*i*_ + ⋯+*β*_*p*_ *n*_*p*_, and thus ord(*s* − *n*_*i*_) + 1 = *β*_1_ + ⋯+(*β*_*i*_ + 1)+⋯+*β*_*p*_ ≤ ord(*s*).

(2) Since *s* − *n*_*i*_ = *α*_1_ *n*_1_ + ⋯+(*α*_*i*_ − 1)*n*_*i*_ + ⋯+*α*_*p*_ *n*_*p*_, we have ord(*s* − *n*_*i*_) ≥ *α*_1_ + ⋯+(*α*_*i*_ − 1)+⋯+*α*_*p*_ = ord(*s*) − 1. Thereby, ord(*s*) − 1 ≤ ord(*s* − *n*_*i*_). Now, by applying the previous item, we conclude that ord(*s* − *n*_*i*_) = ord(*s*) − 1.

In item (2) of the next proposition, it is shown a characterization of PL-semigroups in terms of minimal systems of generators. Thus, we can decide if a numerical semigroup is a PL-semigroup in an easier way.

**Proposition 9**

Let *S* be a numerical semigroup with minimal system of generators given by {*n*_1_,…, *n*_*p*_}. The following conditions are equivalent.

1. *S* is a *PL*-semigroup.
2. If *i*, *j* ∈ {1,…, *p*}, then *n*_*i*_ + *n*_*j*_ + 1 ∈ *S*.
3. If *s* ∈ *S*∖{0, *n*_1_,…, *n*_*p*_}, then *s* + 1 ∈ *S*.
4. If *s* ∈ *S*∖{0}, then *s* + {0,…, *ord*(*s*) − 1}⊆*S*.

**Proof**

(1⇒2) It is an immediate consequence of Theorem 5.

(2⇒3) If *s* ∈ *S*∖{0, *n*_1_,…, *n*_*p*_}, then it is clear that there exist *i*, *j* ∈ {1,…, *p*} and *s*′ ∈ *S* such that *s* = *n*_*i*_ + *n*_*j*_ + *s*′. Thus, *s* + 1 = (*n*_*i*_ + *n*_*j*_ + 1) + *s*′ ∈ *S*.

(3⇒4) We reason by induction over ord(*s*). If ord(*s*) = 1, then the result is trivially true. Now, let us assume that ord(*s*) ≥ 2 and that *α*_1_,…, *α*_*p*_ are nonnegative integers such that *s* = *α*_1_ *n*_1_ + ⋯+*α*_*p*_ *n*_*p*_, ord(*s*) = *α*_1_ + ⋯+*α*_*p*_, and *a*_*i*_ ≠ 0 for some *i* ∈ {1,…, *p*}. By Lemma 8, we know that ord(*s* − *n*_*i*_) = ord(*s*) − 1. Then, by hypothesis of induction, we have that *s* − *n*_*i*_ + {0,…, ord(*s*) − 2}⊆*S*. Therefore, *s* + {0,…, ord(*s*) − 2}⊆*S*. Moreover, (*s* − *n*_*i*_ + ord(*s*) − 2) + *n*_*i*_ + 1 ∈ *S*. Thereby, *s* + {0,…, ord(*s*) − 1}⊆*S*.

(4⇒1) If *x*, *y* ∈ *S*∖{0}, then it is clear that ord(*x* + *y*) ≥ 2. Thus, we get that *x* + *y* + 1 ∈ *S*. By applying Theorem 5, we can conclude that *S* is a PL-semigroup.

**Example 10**

Let *S* = 〈4,5, 6〉; that is, let *S* be the numerical semigroup with minimal system of generators given by {4,5, 6}. It is obvious that 4 + 4 + 1 = 9, 4 + 5 + 1 = 10, 4 + 6 + 1 = 11, 5 + 5 + 1 = 11, 5 + 6 + 1 = 12, and 6 + 6 + 1 = 13 are elements of *S*. Therefore, by applying Proposition 9, we have that *S* is a PL-semigroup.

## 3. The Frobenius Variety of the PL-Semigroups

The following result is straightforward to prove and appears in [5].

**Lemma 11**

Let *S*, *T* be numerical semigroups.

1. *S*∩*T* is a numerical semigroup.
2. If *S* ≠ *ℕ*, then *S* ∪ {F(*S*)} is a numerical semigroup.

Having in mind the definition of Frobenius variety, which was given in the introduction, we get the next result.

**Proposition 12**

The set *𝒮*_*PL*_ = {*S* | *S* is a *PL*-semigroup} is a Frobenius variety.

**Proof**

First of all, let us observe that *ℕ* ∈ *𝒮*_PL_ and, therefore, *𝒮*_PL_ is a nonempty set.

Let *S*, *T* ∈ *𝒮*_PL_. In order to show that *S*∩*T* ∈ *𝒮*_PL_, we are going to use Theorem 5. So, if *x*, *y* ∈ (*S*∩*T*)∖{0}, then *x*, *y* ∈ *S*∖{0} and *x*, *y* ∈ *T*∖{0}. Therefore, *x* + *y* + 1 ∈ *S*∩*T*. Consequently, *S*∩*T* ∈ *𝒮*_PL_.

Now, let *S* ∈ *𝒮*_PL_ such that *S* ≠ *ℕ*. By applying Theorem 5 again, we are going to see that *S* ∪ F(*S*) ∈ *𝒮*_PL_. Let *x*, *y* ∈ (*S* ∪ F(*S*))∖{0}. If *x*, *y* ∈ *S*, then *x* + *y* + 1 ∈ *S*⊆*S* ∪ F(*S*). On the other hand, if F(*S*)∈{*x*, *y*}, then *x* + *y* + 1 > F(*S*) and, thereby, *x* + *y* + 1 ∈ *S*⊆*S* ∪ F(*S*). We conclude that *S* ∪ F(*S*) ∈ *𝒮*_PL_.

A *graph* *G* is a pair (*V*, *E*), where *V* is a nonempty set and *E* is a subset of {(*v*, *w*) ∈ *V* × *V* | *v* ≠ *w*}. The elements of *V* are called *vertices* of *G* and the elements of *E* are called *edges* of *G*. A *path (of length n*) connecting the vertices *x* and *y* of *G* is a sequence of different edges of the form (*v*_0_, *v*_1_), (*v*_1_, *v*_2_),…, (*v*_*n*−1_, *v*_*n*_) such that *v*_0_ = *x* and *v*_*n*_ = *y*.

We say that a graph *G* is a *tree* if there exist a vertex v* (known as the *root* of *G*) such that for every other vertex *x* of *G*, there exists a unique path connecting *x* and v*. If (*x*, *y*) is an edge of the tree, then we say that *x* is a *son* of *y*.

We define the graph G(*𝒮*_*PL*_) in the following way:

- *𝒮*_*PL*_ is the set of vertices of G(*𝒮*_PL_),
- (*S*, *S*′) ∈ *𝒮*_*PL*_ × *𝒮*_*PL*_ is an edge of G(*𝒮*_*PL*_) if *S*′ = *S* ∪ {F(*S*)}.

As a consequence of [3, Proposition 24, Theorem 27], we have the next result.

**Theorem 13**

The graph G(*𝒮*_*PL*_) is a tree with root equal to *ℕ*. Moreover, the sons of a vertex *S* ∈ *𝒮*_*PL*_ are *S*∖{*x*_1_},…, *S*∖{*x*_*r*_}, where *x*_1_,…, *x*_*r*_ are the minimal generators of *S* that are greater than F(*S*) and such that *S*∖{*x*_1_},…, *S*∖{*x*_*r*_} ∈ *𝒮*_*PL*_.

Let us observe that if *S* is a numerical semigroup and *x* ∈ *S*, then *S*∖{*x*} is a numerical semigroup if and only if *x* is a minimal generator of *S*. In fact, *S*∖{*x*} is a numerical semigroup whenever *x* ∈ *S*∖{0} and *x* ≠ *y* + *z* for all *y*, *z* ∈ *S*∖{0}. As a consequence, if we denote by msg(*S*) the minimal system of generators of *S*, then msg(*S*) = (*S*∖{0})∖((*S*∖{0})+(*S*∖{0})) (see [5, Lemma 2.3] for other proof of this result). In the following proposition we obtain an analogous for PL-semigroups of the first commented fact in this paragraph.

**Proposition 14**

Let *S* be a *PL*-semigroup and let *x* be a minimal generator of *S*. Then *S*∖{*x*} is a PL-semigroup if and only if *x* − 1 ∈ {0} ∪ (*ℕ*∖*S*) ∪ (msg(*S*)).

**Proof**

*(Necessity).* If *x* − 1 ∉ {0} ∪ (*ℕ*∖*S*) ∪ (msg(*S*)), then *x* − 1 ∈ *S*∖({0} ∪ (msg(*S*))). Accordingly, there exist *y*, *z* ∈ *S*∖{0} such that *x* − 1 = *y* + *z*. In fact, it is clear that *y*, *z* ∈ *S*∖{*x*, 0}. Therefore, by applying Theorem 5 and that *S*∖{*x*} is a PL-semigroup, we have *x* = *y* + *z* + 1 ∈ *S*∖{*x*}, which is a contradiction.

*(Sufficiency).* Let *y*, *z* ∈ *S*∖{*x*, 0}. Since *S* is a PL-semigroup, by Theorem 5 we have *y* + *z* + 1 ∈ *S*. As *x* − 1 ∈ {0} ∪ (*ℕ*∖*S*) ∪ (msg(*S*)), we deduce that *y* + *z* + 1 ≠ *x*. Thus *y* + *z* + 1 ∈ *S*∖{*x*}. By applying Theorem 5 again, we conclude that *S*∖{*x*} is a PL-semigroup.

As a consequence of the previous proposition, we have the next result.

**Corollary 15**

Let *S* be a *PL*-semigroup such that *S* ≠ *ℕ*, and let *x* be a minimal generator of *S* greater than F(*S*). Then *S*∖{*x*} is a *PL*-semigroup if and only if *x* − 1 ∈ msg(*S*) ∪ {F(*S*)}.

By applying Theorem 13 together with Corollary 15, we can get the sons of a vertex of G(*𝒮*_PL_) as is shown in the following example.

**Example 16**

It is clear that *S* = 〈4,6, 7,9〉 is a PL-semigroup with Frobenius number equal to 5. From Theorem 13 and Corollary 15, we deduce that the sons of *S* are *S*∖{6} = 〈4,7, 9,10〉 and *S*∖{7} = 〈4,6, 9,11〉.

**Figure 1:**
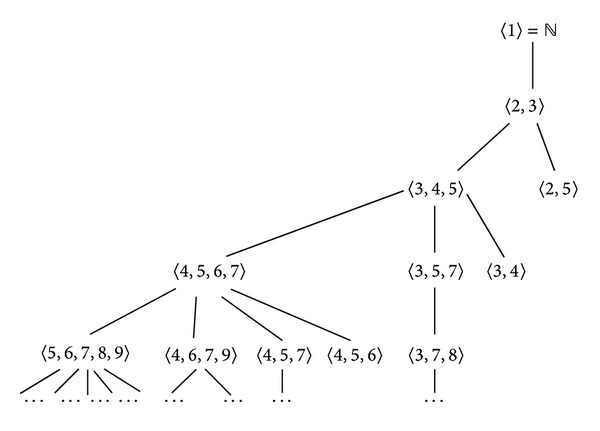


Let us observe that we can build recursively a tree, from the root, if we know the sons of each vertex. Therefore, we can build the tree G(*𝒮*_PL_) such as it is shown in Figure 1.

In order to have an easier making of the tree G(*𝒮*_PL_), we are going to study the relation between the minimal generators of a numerical semigroup *S* and the minimal generators of *S*∖{*x*}, where *x* is a minimal generator of *S* that is greater than F(*S*). First of all, let us observe that if *S* is minimally generated by {*m*, *m* + 1,…, 2*m* − 1} (i.e., *S* = {0, *m*, →}), then *S*∖{*m*} = {0, *m* + 1, →} is minimally generated by {*m* + 1, *m* + 2,…, 2*m* + 1}. In other case we have the following result.

**Proposition 17**

Let *S* be a numerical semigroup with msg(*S*) = {*n*_1_,…, *n*_*p*_}. If m(*S*) = *n*_1_ < *n*_*p*_ and *n*_*p*_ > F(S), then *S*∖{*n*_*p*_} = 〈*n*_1_,…, *n*_*p*−1_, *n*_*p*_ + *n*_1_〉.

**Proof**

Let us take *i* ∈ {2,…, *p*}. Since *n*_*p*_ > F(*S*) and *n*_1_ < *n*_*i*_, we have that *n*_*p*_ + *n*_*i*_ − *n*_1_ ∈ *S*. Thus, *n*_*p*_ + *n*_*i*_ − *n*_1_ = *α*_1_ *n*_1_ + ⋯+*α*_*p*_ *n*_*p*_ for some *α*_1_,…, *α*_*p*_ ∈ *ℕ*. Thereby, *n*_*p*_ + *n*_*i*_ = (*α*_1_ + 1)*n*_1_ + ⋯+*α*_*p*_ *n*_*p*_. By applying that {*n*_1_,…, *n*_*p*_} is a minimal system of generators, we have that *α*_*p*_ = 0. Therefore, *n*_*p*_ + *n*_*i*_ ∈ 〈*n*_1_,…, *n*_*p*−1_〉. In particular, 2*n*_*p*_ ∈ 〈*n*_1_,…, *n*_*p*−1_〉.

Now, let *s* ∈ *S*∖{*n*_*p*_}. Then *s* ∈ *S* and, thus, there exist *β*_1_,…, *β*_*p*_ ∈ *ℕ* such that *s* = *β*_1_ *n*_1_ + ⋯+*β*_*p*_ *n*_*p*_. Since 2*n*_*p*_ ∈ 〈*n*_1_,…, *n*_*p*−1_〉, we can assume that *β*_*p*_ ∈ {0,1}. On the one hand, if *β*_*p*_ = 0, then *s* ∈ 〈*n*_1_,…, *n*_*p*−1_〉. On the other hand, if *β*_*p*_ = 1, then there exists *i* ∈ {1,…, *p* − 1} such that *β*_*i*_ ≠ 0. If *i* = 1, then it is obvious that *s* ∈ 〈*n*_1_,…, *n*_*p*−1_, *n*_*p*_ + *n*_1_〉. And if *i* ≠ 1, since *n*_*p*_ + *n*_*i*_ ∈ 〈*n*_1_,…, *n*_*p*−1_〉, we have that *s* ∈ 〈*n*_1_,…, *n*_*p*−1_〉. In any case, we conclude that *S*∖{*n*_*p*_} = 〈*n*_1_,…, *n*_*p*−1_, *n*_*p*_ + *n*_1_〉.

**Corollary 18**

Let *S* be a numerical semigroup with msg(*S*) = {*n*_1_,…, *n*_*p*_}. If m(*S*) = *n*_1_ < *n*_*p*_ and *n*_*p*_ > F(*S*), then

(3)  
msg
(S∖{np})={{n1,…,np−1},if there exists i∈{2,…,p−1}such that np+n1−ni∈S;{n1,…,np−1,np+n1},in other case.

**Proof**

From Proposition 17 we deduce that msg(*S*∖{*n*_*p*_}) is {*n*_1_,…, *n*_*p*−1_} or {*n*_1_,…, *n*_*p*−1_, *n*_*p*_ + *n*_1_}. In addition, msg(*S*∖{*n*_*p*_}) = {*n*_1_,…, *n*_*p*−1_} if and only if *n*_*p*_ + *n*_1_ ∈ 〈*n*_1_,…, *n*_*p*−1_〉.

If *n*_*p*_ + *n*_1_ ∈ 〈*n*_1_,…, *n*_*p*−1_〉, then there exist *α*_1_,…, *α*_*p*−1_ ∈ *ℕ* such that *n*_*p*_ + *n*_1_ = *α*_1_ *n*_1_ + ⋯+*α*_*p*−1_ *n*_*p*−1_. Since {*n*_1_,…, *n*_*p*_} is a minimal system of generators, we get that *α*_1_ = 0. Thus, there exists *i* ∈ {2,…, *p* − 1} such that *α*_*i*_ ≠ 0. Consequently, *n*_*p*_ + *n*_1_ − *n*_*i*_ ∈ *S*.

Conversely, if there exists *i* ∈ {2,…, *p* − 1} such that *n*_*p*_ + *n*_1_ − *n*_*i*_ ∈ *S*, then *n*_*p*_ + *n*_1_ − *n*_*i*_ = *β*_1_ *n*_1_ + ⋯+*β*_*p*_ *n*_*p*_ for some *β*_1_,…, *β*_*p*_ ∈ *ℕ*. Thereby, *n*_*p*_ + *n*_1_ = *β*_1_ *n*_1_ + ⋯+(*β*_*i*_ + 1)*n*_*i*_ + ⋯+*β*_*p*_ *n*_*p*_. Since {*n*_1_,…, *n*_*p*_} is a minimal system of generators, we have that *β*_*p*_ = 0 and, therefore, *n*_*p*_ + *n*_1_ ∈ 〈*n*_1_,…, *n*_*p*−1_〉.

We finish this section with an illustrative example about the above corollary.

**Example 19**

Let *S* be the numerical semigroup with msg(*S*) = {3,5, 7}. It is obvious that F(*S*) = 4. By Proposition 17, we know that *S*∖{5} = 〈3,7, 8〉. In addition, 8 − 7 = 1 ∉ *S*. Thereby, applying Corollary 18, we have that msg(*S*∖{5}) = {3,7, 8}. On the other hand, applying Proposition 17 again, we have that *S*∖{7} = 〈3,5, 10〉. Finally, since 10 − 5 = 5 ∈ *S*, we conclude that msg(*S*∖{7}) = {3,5}.

## 4. PL-Semigroups with a Fixed Multiplicity

Let *m* be a positive integer. We will denote by Δ(*m*) = {0, *m*, →}. It is clear that Δ(*m*) is the greatest (with respect to set inclusion) PL-semigroup with multiplicity *m*. Our first aim in this section will be to show that there also exists the smallest (with respect to set inclusion) PL-semigroup with multiplicity *m*.

As an immediate consequence of item (4) in Proposition 9 we have the next result.

**Lemma 20**

If *S* is a *PL*-semigroup, *m* ∈ *S*∖{0}, and *k* ∈ *ℕ*∖{0}, then *km* + *i* ∈ *S* for all *i* ∈ {0,…, *k* − 1}.

**Proposition 21**

Let *m* ∈ *ℕ*∖{0}. Then the numerical semigroup generated by {(*i* + 1)*m* + *i* | *i* ∈ {0,…, *m* − 1}} is the smallest (with respect to set inclusion) *PL*-semigroup with multiplicity *m*.

**Proof**

Let *S* = 〈*m*, 2*m* + 1,…, *m* ^2^ + (*m* − 1)〉. From Lemma 20, we know that any PL-semigroup with multiplicity *m* has to contain *S*. In order to conclude the proof, we will show that *S* is a PL-semigroup. For this purpose, since {(*i* + 1)*m* + *i* | *i* ∈ {0,…, *m* − 1}} is a system of generators of *S*, it will be enough to check item (2) of Proposition 9; that is, if *i*, *j* ∈ {0,…, *m* − 1}, then (*i* + 1)*m* + *i* + (*j* + 1)*m* + *j* + 1 ∈ *S*. We distinguish two cases.

1. If *i* + *j* + 1 ≤ *m* − 1, then (*i* + 1)*m* + *i* + (*j* + 1)*m* + *j* + 1 = (*i* + *j* + 2)*m* + (*i* + *j* + 1) ∈ *S*.
2. If *i* + *j* + 1 ≥ *m*, then (*i* + 1)*m* + *i* + (*j* + 1)*m* + *j* + 1 = (*i* + *j* + 2)*m* + (*i* + *j* + 1) = (*m* + 1)*m* + (*i* + *j* − *m* + 2)*m* + (*i* + *j* − *m* + 1) ∈ *S*.

We will denote by Θ(*m*) = 〈*m*, 2*m* + 1,…, *m* ^2^ + (*m* − 1)〉 and by *𝒮*_PL_(*m*) the set of all PL-semigroups with multiplicity equal to *m*. Let us recall that Δ(*m*) = max⁡(*𝒮*_PL_(*m*)) and Θ(*m*) = min⁡(*𝒮*_PL_(*m*)).

As an application of the above comment, we have the next result.

**Corollary 22**

The set *𝒮*_PL_(*m*) is finite.

**Proof**

If *S* ∈ *𝒮*_PL_(*m*), then Θ(*m*)⊆*S*⊆Δ(*m*). Since Δ(*m*) and Θ(*m*) are numerical semigroups, we have that Δ(*m*)∖Θ(*m*) is finite. Consequently, *𝒮*_PL_(*m*) is also finite.

**Remark 23**

The previous result can be considered a particular case of [6, Theorem 6.6].

Now we are interested in computing the Frobenius number and the genus of Θ(*m*). For that, several concepts and results are introduced.

If *S* is a numerical semigroup and *m* ∈ *S*∖{0}, then the *Apéry set* of *m* in *S* (see [9]) is Ap(*S*, *m*) = {*s* ∈ *S* | *s* − *m* ∉ *S*}. It is clear (see for instance [5, Lemma 2.4]) that Ap(*S*, *m*) = {*ω*(0) = 0, *ω*(1),…, *ω*(*m* − 1)}, where *ω*(*i*) is, for each *i* ∈ {0,…, *m* − 1}, the least element of *S* that is congruent with *i* modulo *m*.

The next result is [5, Proposition 2.12].

**Lemma 24**

Let *S* be a numerical semigroup and let *m* ∈ *S*∖{0}. Then

1. F(*S*) = max⁡(Ap(*S*, *m*)) − *m*,
2. g(*S*) = (1/*m*)(∑_*w*∈*Ap*(*S*,*m*)_ *w*) − ((*m* − 1)/2).

If *a*, *b* are integers with *b* ≠ 0, we denote by *a* mod⁡ *b* the remainder of the division of *a* by *b*. The following result is [5, Proposition 3.5].

**Lemma**

Let *m* ∈ *ℕ*∖{0} and let *X* = {*ω*(0) = 0, *ω*(1),…, *ω*(*m* − 1)}⊆*ℕ* such that, for each *i* ∈ {1,…, *m* − 1}, *ω*(*i*) is congruent with *i* modulo *m*. Let *S* be the numerical semigroup generated by *X* ∪ {*m*}. The following conditions are equivalent.

1. Ap(*S*, *m*) = *X*.
2. *ω*(*i*) + *ω*(*j*) ≥ *ω*((*i* + *j*)mod⁡*m*) for all *i*, *j* ∈ {1,…, *m* − 1}.

**Proposition 26**

If *m* ∈ *ℕ*∖{0}, then

(4)  Ap(Θ(m),m)={ω(0)=0,ω(1)=2m+1,…,ω(m−1)=m2+m−1}.

**Proof**

It is clear that *ω*(*i*) = (*i* + 1)*m* + *i* is congruent with *i* modulo *m* for all *i* ∈ {1,…, *m* − 1}. Let us see now that, if *i*, *j* ∈ {1,…, *m* − 1}, then *ω*(*i*) + *ω*(*j*) ≥ *ω*((*i* + *j*)mod⁡ *m*). Indeed, *ω*(*i*) + *ω*(*j*) = (*i* + 1)*m* + *i* + (*j* + 1)*m* + *j* > (*i* + *j* + 1)*m* + (*i* + *j*)≥((*i* + *j*)mod⁡ *m* + 1)*m* + (*i* + *j*)mod⁡ *m* = *ω*((*i* + *j*)mod⁡ *m*). The proof follows from Lemma 25.

**Corollary 27**

If *m* ∈ *ℕ*∖{0,1}, then

1. F(Δ(*m*)) = *m* − 1,
2. g(Δ(*m*)) = *m* − 1,
3. F(Θ(*m*)) = *m* ^2^ − 1,
4. g(Θ(*m*)) = (*m* − 1)(*m* + 2)/2.

**Proof**

Items (1) and (2) are trivial. On the other hand, items (3) and (4) are immediate consequences of Lemma 24 and Proposition 26.

**Remark 28**

The numerical semigroup Θ(*m*) can be rewritten as

(5) Θ(m)={m,m+(m+1),m+2(m+1),…,m+(m−1)(m+1)}.

Thus Θ(*m*) is a numerical semigroup generated by an *arithmetic sequence* with first term *m* and common difference *m* + 1 (see [2, 10]).

If *S* is a numerical semigroup, then the cardinality of the minimal system of generators of *S* is called the *embedding dimension* of *S* and is denoted by e(*S*). It is well known (see [5, Proposition 2.12]) that e(*S*) ≤ m(*S*). We say that a numerical semigroup *S* has maximal embedding dimension if e(*S*) = m(*S*). It is clear that {*m*, *m* + 1,…, 2*m* − 1} is the minimal system of generators of Δ(*m*). Therefore, Δ(*m*) is a numerical semigroup with maximal embedding dimension. Now we will show that Θ(*m*) has also maximal embedding dimension.

The next result is [5, Corollary 3.6].

**Lemma 29**

Let *S* be a numerical semigroup with multiplicity *m* and assume that Ap(*S*, *m*) = {*ω*(0) = 0, *ω*(1),…, *ω*(*m* − 1)}. Then *S* has maximal embedding dimension if and only if *ω*(*i*) + *ω*(*j*) > *ω*((*i* + *j*)mod⁡ *m*) for all *i*, *j* ∈ {1,…, *m* − 1}.

Let us observe that, in the proof of Proposition 26, we have shown that *ω*(*i*) + *ω*(*j*) > *ω*((*i* + *j*)mod⁡ *m*) for all *i*, *j* ∈ {1,…, *m* − 1}. Therefore, by applying Lemma 29, we get the following result.

**Corollary 30**

If *m* ∈ *ℕ*∖{0}, then Θ(*m*) is a numerical semigroup with maximal embedding dimension.

As a consequence of this corollary, we have that {*m*, 2*m* + 1,…, *m* ^2^ + *m* − 1} is the minimal system of generators of Θ(*m*).

Now, we want to show orderly the elements of *𝒮*_PL_(*m*). Thus, we define the graph G(*𝒮*_PL_(*m*)) in the following way:

- *𝒮*_PL_(*m*) is the set of vertices of G(*𝒮*_PL_(*m*));
- (*S*, *S*′) ∈ *𝒮*_PL_(*m*) × *𝒮*_PL_(*m*) is an edge of G(*𝒮*_PL_(*m*)) if *S*′ = *S* ∪ {F(*S*)}.

The next result is analogous to Theorem 13.

**Theorem 31**

The graph G(*𝒮*_*PL*_(*m*)) is a tree with root equal to Δ(*m*). Moreover, the sons of a vertex *S* ∈ *𝒮*_*PL*_(*m*) are *S*∖{*x*_1_},…, *S*∖{*x*_*r*_}, with {*x*_1_,…, *x*_*r*_} = {*x* ∈ msg(*S*) | *x* ≠ *m*, *x* > F(*S*),  *and* *S*∖{*x*} ∈ *𝒮*_*PL*_}.

By applying Theorem 31 and Corollaries 15 and 18, we can get easily G(*𝒮*_PL_(*m*)) such as is shown in the next example.

**Example 32**

We are going to depict G(*𝒮*_PL_(3)), that is, the tree of the PL-semigroups with multiplicity equal to 3.

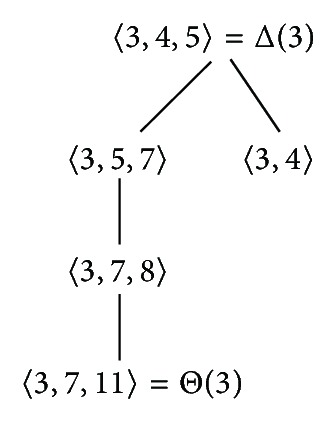


If *T* = (*V*, *E*) is a tree, then the *height* of *T* is the maximum of the lengths of the paths that connect each vertex with the root. Let us observe that the height of G(*𝒮*_PL_(3)) is 3. In general, the height of the tree G(*𝒮*_PL_(*m*)) is equal to

(6) g(Θ(m))−g(Δ(m))=(m−1)(m+2)2−(m−1)=(m−1)m2.

Let us study now the possible values of the Frobenius number and the genus for PL-semigroups with multiplicity *m*.

**Proposition 33**

If *m* ∈ *ℕ*∖{0}, then

1. {g(*S*) | *S* ∈ *𝒮*_PL_(*m*)} = {*x* ∈ *ℕ* | *m* − 1 ≤ *x* ≤ (*m* − 1)(*m* + 2)/2};
2. {F(*S*) | *S* ∈ *𝒮*_PL_(*m*)} = (Δ(*m*)∖Θ(*m*)) ∪ {*m* − 1}.

**Proof**

Let us assume that Δ(*m*)∖Θ(*m*) = {*x*_1_ > *x*_2_ > ⋯>*x*_*ρ*_}.

(1) By Corollary 27, we know that

(7) $$\begin{array}{c} \left\{\text{g}\left(S\right)\;|\;S\in {𝒮}_{\text{PL}}\left(m\right)\right\}\subseteq \left\{m-1,\ldots ,\frac{\left(m-1\right)\left(m+2\right)}{2}\right\}. \end{array}$$

For the opposite inclusion it is enough to observe that Θ(*m*)∪{*x*_*i*_, →} ∈ *𝒮*_PL_(*m*) and that g(Θ(*m*)∪{*x*_*i*_, →}) = (*m* − 1)(*m* + 2)/2 − *i*.

(2) It is clear that Θ(*m*)∪{*x*_*i*_ + 1, →} ∈ *𝒮*_PL_(*m*) with Frobenius number equal to *x*_*i*_. Thus, (Δ(*m*)∖Θ(*m*)) ∪ {*m* − 1}⊆{F(*S*) | *S* ∈ *𝒮*_PL_(*m*)}. For the other inclusion, let us take *S* ∈ *𝒮*_PL_(*m*) such that *S* ≠ Δ(*m*). Then F(*S*) > *m* and, thereby, F(*S*) ∈ Δ(*m*). Since Θ(*m*)⊆*S*, we have F(*S*) ∉ Θ(*S*). Therefore, we conclude that F(*S*) ∈ Δ(*m*)∖Θ(*S*).

**Example 34**

By Proposition 33, {g(*S*) | *S* ∈ *𝒮*_PL_(3)} = {2,3, 4,5}. Since Δ(3) = 〈3,4, 5〉 and Θ(3) = 〈3,7, 11〉, we have that (Δ(3)∖Θ(3)) ∪ {2} = {2,4, 5,8}. Therefore, by applying Proposition 33 again, we conclude that {F(*S*) | *S* ∈ *𝒮*_PL_(3)} = {2,4, 5,8}.

## 5. The Smallest PL-Semigroup That Contains a Given Set of Positive Integers

Let us observe that, in general, the infinite intersection of elements of *𝒮*_PL_ is not a numerical semigroup. For instance, ⋂_*n*∈*ℕ*_{0, *n*, →} = {0}. On the other hand, it is clear that the (finite or infinite) intersection of numerical semigroups is always a submonoid of (*ℕ*, +).

Let *M* be a submonoid of (*ℕ*, +). We will say that *M* is a *𝒮*_PL_ *-monoid* if it can be expressed like the intersection of elements of *𝒮*_PL_.

The next lemma has an immediate proof.

**Lemma 35**

The intersection of *𝒮*_*PL*_-monoids is a *𝒮*_*PL*_-monoid.

In view of this result, we can give the following definition.

**Definition 36**

Let *X* be a subset of *ℕ*. The *𝒮*_PL_-monoid generated by *X* (denoted by *𝒮*_PL_(*X*)) is the intersection of all *𝒮*_PL_-monoids containing *X*.

If *M* = *𝒮*_PL_(*X*), then we will say that *X* is a *𝒮*_PL_ *-system of generators* of *M*. In addition, if no proper subset of *X* is a *𝒮*_PL_-system of generators of *M*, then we will say that *X* is a *minimal 𝒮*_PL_ *-system of generators* of *M*.

Let us recall that, by Proposition 12, we know that *𝒮*_*PL*_ is a Frobenius variety. Therefore, by applying [3, Corollary 19], we have the next result.

**Proposition 37**

Every *𝒮*_*PL*_-monoid has a unique minimal *𝒮*_*PL*_-system of generators, which in addition is finite.

The proof of the following lemma is also immediate.

**Lemma 38**

If *X*⊆*ℕ*, then *𝒮*_*PL*_(*X*) is the intersection of all *PL*-semigroups that contain *X*.

**Proposition 39**

If *X* is a nonempty subset of *ℕ*∖{0}, then *𝒮*_*PL*_(*X*) is a *PL*-semigroup.

**Proof**

We know that *𝒮*_PL_(*X*) is a submonoid of (*ℕ*, +). Therefore, in order to show that *𝒮*_PL_(*X*) is a numerical semigroup, it will be enough to see that *ℕ*∖*𝒮*_PL_(*X*) is a finite set.

Let *x* ∈ *X*. If *S* is a PL-semigroup containing *X*, then (by Theorem 5) we know that {*x*, 2*x* + 1}⊆*S* and, in this way, 〈*x*, 2*x* + 1〉⊆*S*. From Lemma 38, we have that 〈*x*, 2*x* + 1〉⊆*𝒮*_PL_(*X*). Since gcd{*x*, 2*x* + 1} = 1, we get that 〈*x*, 2*x* + 1〉 is a numerical semigroup and, thus, *ℕ*∖〈*x*, 2*x* + 1〉 is finite. Consequently, *ℕ*∖*𝒮*_PL_(*X*) is finite.

Now, let us see that *𝒮*_PL_(*X*) is a PL-semigroup. Let *x*, *y* ∈ *𝒮*_PL_(*X*)∖{0}. If *S* is a PL-semigroup containing *X*, from Lemma 38, we deduce that *x*, *y* ∈ *S*∖{0} and from Theorem 5, we have that *x* + *y* + 1 ∈ *S*. By applying again Lemma 38, we have that *x* + *y* + 1 ∈ *𝒮*_PL_(*X*). Therefore, by applying Theorem 5 once more, we can assert that *𝒮*_PL_(*X*) is a PL-semigroup.

**Remark 40**

Let us observe that, in general, Proposition 39 is not true for Frobenius varieties. In fact, let *𝒮* be the set of all numerical semigroups. It is clear that *𝒮* is a Frobenius variety. If we take *X* = {2}, then the intersection of all elements of *𝒮* containing {2} is exactly *M* = 〈2〉, which is not a numerical semigroup.

The next result will be key for our last purpose in this section.

**Theorem 41**

*𝒮*_*PL*_ = {*𝒮*_*PL*_(*X*) | *X* *is* *a* *nonempty* *finite* *subset* *of* *ℕ*∖{0}}.

**Proof**

By Proposition 39, we have that

(8) $$\begin{array}{c} \left\{{𝒮}_{\text{PL}}\left(X\right)\;|\;X\;\text{is}\;\text{a}\;\text{nonempty}\;\text{finite}\;\text{subset}\;\text{of}\;\mathbb{N}\setminus \left\{0\right\}\right\}\subseteq {𝒮}_{\text{PL}}. \end{array}$$

For the other inclusion it is enough to observe that if *S* ∈ *𝒮*_PL_, then (by Proposition 37) there exists a nonempty finite subset *X* of *ℕ*∖{0} such that *S* = *𝒮*_PL_(*X*).

Since *𝒮*_PL_ is a Frobenius variety, by applying [3, Proposition 24], we get the next result.

**Proposition 42**

Let *M* be a *𝒮*_*PL*_-monoid and let *x* ∈ *M*. Then *M*∖{*x*} is a *𝒮*_*PL*_-monoid if and only if *x* belongs to the minimal *𝒮*_*PL*_-system of generators of *M*.

As an immediate consequence of this proposition we have the following result.

**Corollary 43**

Let *X* be a nonempty subset of *ℕ*∖{0}. Then the minimal *𝒮*_*PL*_-system of generators of *𝒮*_*PL*_(*X*) is {*x* ∈ *X* | *𝒮*_*PL*_(*X*)∖{*x*} is a PL-semigroup}.

**Example 44**

By Proposition 21, *S* = 〈3,7, 11〉 is a PL-semigroup. By applying Proposition 14, we easily deduce that

(9) $$\begin{array}{c} \left\{x\in \left\{3,7,11\right\}\;|\;S\setminus \left\{x\right\}\;\text{is}\;\text{a}\;\text{PL-semigroup}\right\}=\left\{3\right\}. \end{array}$$

Therefore, *S* = *𝒮*_PL_({3}) and {3} is the minimal *𝒮*_PL_-system of generators of *S*.

Now we want to describe *𝒮*_PL_(*X*) when *X* is a fixed nonempty finite set of positive integers. Let us observe that by Theorem 41, we know that every PL-semigroup can be obtained in this way.

Let *n*_1_,…, *n*_*p*_ be positive integers. We will denote by *S*(*n*_1_,…, *n*_*p*_) the set {*α*_1_ *n*_1_ + ⋯+*α*_*p*_ *n*_*p*_ + *r* | *r*, *α*_1_,…, *α*_*p*_ ∈ *ℕ*, *r* < *α*_1_ + ⋯+*α*_*p*_} ∪ {0}. Our next purpose will be to show that *S*(*n*_1_,…, *n*_*p*_) = *𝒮*_PL_({*n*_1_,…, *n*_*p*_}).

**Lemma 45**

Let *S* be a numerical semigroup, let *s*_1_,…, *s*_*t*_ ∈ *S*∖{0}, and let *α*_1_,…, *α*_*t*_ ∈ *ℕ*. Then ord(*α*_1_ *s*_1_ + ⋯+*α*_*t*_ *s*_*t*_) ≥ *α*_1_ + ⋯+*α*_*t*_.

**Proof**

Let {*n*_1_,…, *n*_*p*_} be the minimal system of generators of *S*. Then, for each *i* ∈ {1,…, *t*}, there exist *β*_*i*1_,…, *β*_*ip*_ ∈ *ℕ* such that *s*_*i*_ = *β*_*i*1_ *n*_1_ + ⋯+*β*_*ip*_ *n*_*p*_. Moreover, since *s*_*i*_ ≠ 0, we have that *β*_*i*1_ + ⋯+*β*_*ip*_ ≥ 1. Thus,

(10) α1s1+⋯+αtst =α1(β11n1+⋯+β1pnp)+⋯+αt(βt1n1+⋯+βtpnp) =(α1β11+⋯+αtβt1)n1+⋯+(α1β1p+⋯+αtβtp)np.

Therefore,

(11) ord(α1s1+⋯+αtst) ≥(α1β11+⋯+αtβt1)+⋯+(α1β1p+⋯+αtβtp) =α1(β11+⋯+β1p)+⋯+αt(βt1+⋯+βtp) ≥α1+⋯+αt.

**Theorem 46**

If *n*_1_,…, *n*_*p*_ are positive integers, then *S*(*n*_1_,…, *n*_*p*_) is the smallest (with respect to set inclusion) *PL*-semigroup containing {*n*_1_,…, *n*_*p*_}.

**Proof**

We divide the proof into five steps.

- Let us see that if *x*, *y* ∈ *S*(*n*_1_,…, *n*_*p*_)∖{0}, then *x* + *y* ∈ *S*(*n*_1_,…, *n*_*p*_). In effect, we know that there exist *α*_1_,…, *α*_*p*_, *β*_1_,…, *β*_*p*_, *r*, *r*′ nonnegative integers such that *x* = *α*_1_ *n*_1_ + ⋯+*α*_*p*_ *n*_*p*_ + *r*, *y* = *β*_1_ *n*_1_ + ⋯+*β*_*p*_ *n*_*p*_ + *r*′, *r* < *α*_1_ + ⋯+*α*_*p*_, and *r*′ < *β*_1_ + …+*β*_*p*_. Therefore, *x* + *y* = (*α*_1_ + *β*_1_)*n*_1_ + ⋯+(*α*_*p*_ + *β*_*n*_)*n*_*p*_ + *r* + *r*′ with *r* + *r*′ < (*α*_1_ + *β*_1_)+⋯+(*α*_*p*_ + *β*_*n*_). Consequently, *x* + *y* ∈ *S*(*n*_1_,…, *n*_*p*_).
- Let us see that *ℕ*∖*S*(*n*_1_,…, *n*_*p*_) is finite. Since *n*_1_ = 1 · *n*_1_ + 0 · *n*_2_ + ⋯+0 · *n*_*p*_ + 0 and 2*n*_1_ + 1 = 2 · *n*_1_ + 0 · *n*_2_ + ⋯+0 · *n*_*p*_ + 1, we have that *n*_1_, 2*n*_1_ ∈ *S*(*n*_1_,…, *n*_*p*_). By applying the first step, we get that 〈*n*_1_, 2*n*_1_〉⊆*S*(*n*_1_,…, *n*_*p*_). Using the same reasoning as we did in the proof of Proposition 39, we have the result.
- From the previous steps, we know that *S*(*n*_1_,…, *n*_*p*_) is a numerical semigroup. Let us see now that *S*(*n*_1_,…, *n*_*p*_) is a PL-semigroup. In order to do that, it is enough (by Theorem 5) to show that, if *x*, *y* ∈ *S*(*n*_1_,…, *n*_*p*_)∖{0}, then *x* + *y* + 1 ∈ *S*(*n*_1_,…, *n*_*p*_). Indeed, arguing as in the first step, we have that *x* + *y* + 1 = (*α*_1_ + *β*_1_)*n*_1_ + ⋯+(*α*_*p*_ + *β*_*n*_)*n*_*p*_ + *r* + *r*′ + 1 with *r* + *r*′ + 1 < (*α*_1_ + *β*_1_)+⋯+(*α*_*p*_ + *β*_*n*_). Therefore, *x* + *y* + 1 ∈ *S*(*n*_1_,…, *n*_*p*_).
- Following the proof of the second step, it is clear that {*n*_1_,…, *n*_*p*_}⊆*S*(*n*_1_,…, *n*_*p*_).
- Finally, let us see that *S*(*n*_1_,…, *n*_*p*_) is the smallest PL-semigroup that contains {*n*_1_,…, *n*_*p*_}. In fact, we will show that if *T* is PL-semigroup containing {*n*_1_,…, *n*_*p*_}, then *S*(*n*_1_,…, *n*_*p*_)⊆*T*. Thus, let *x* ∈ *S*(*n*_1_,…, *n*_*p*_)∖{0}. Then there exist *α*_1_,…, *α*_*p*_, *r* ∈ *ℕ* such that *x* = *α*_1_ *n*_1_ + ⋯+*α*_*p*_ *n*_*p*_ + *r* with *r* < *α*_1_ + ⋯+*α*_*p*_. Since {*n*_1_,…, *n*_*p*_}⊆*T*, by Proposition 9, we have that *α*_1_ *n*_1_ + ⋯+*α*_*p*_ *n*_*p*_ + {0,…, ord(*α*_1_ *n*_1_ + ⋯+*α*_*p*_ *n*_*p*_) − 1}⊆*T*. By applying Lemma 45, we have that *r* < ord(*α*_1_ *n*_1_ + ⋯+*α*_*p*_ *n*_*p*_) and, therefore, *x* ∈ *T*.

In this way, we have proved the statement.

The next result is an immediate consequence of the previous theorem.

**Corollary 47**

If *n*_1_,…, *n*_*p*_ are positive integers, then *𝒮*_PL_({*n*_1_,…, *n*_*p*_}) = *S*(*n*_1_,…, *n*_*p*_).

We finish this section with an example that illustrates its content.

**Example 48**

It is clear that *S*(4,7) = {0,4, 7,8, 9,11, →} = 〈4,7, 9〉. Therefore, *𝒮*_PL_({4,7}) = 〈4,7, 9〉.

## References

[1] Lothaire M (2005). *Applied Combinatorics on Words*.

[2] Ramírez Alfonsín JL (2005). *The Diophantine Frobenius Problem*.

[3] Rosales JC (2008). Families of numerical semigroups closed under finite intersections and for the Frobenius number. *Houston Journal of Mathematics*.

[4] Rosales JC, Branco MB, Torrão D Bracelet monoids and numerical semigroups.

[5] Rosales JC, García-Sánchez PA (2009). *Numerical Semigroups*.

[6] Bras-Amorós M, García-Sánchez PA, Vico-Oton A (2013). Nonhomogeneous patterns on numerical semigroups. *International Journal of Algebra and Computation*.

[7] Stokes K, Bras-Amorós M Linear, non-homogeneous, symmetric patterns and prime power generators in numerical semigroups associated to combinatorial
congurations.

[8] Bryant L (2010). Goto numbers of a numerical semigroup ring and the gorensteiness of associated graded rings. *Communications in Algebra*.

[9] Apéry R (1946). Sur les branches superlinéaires des courbes algébriques. *Comptes Rendus de l'Académie des Sciences*.

[10] Roberts JB (1956). Note on linear forms. *Proceedings of the American Mathematical Society*.

